# Supplementary material for: Health seeking behavior after the 2013–16 Ebola epidemic: Lassa fever as a metric of persistent changes in Kenema District, Sierra Leone
Source: PLoS Negl Trop Dis. 2021 Jul 14;15(7):e0009576. doi: 10.1371/journal.pntd.0009576 (PMC8312964; doi:10.1371/journal.pntd.0009576)
Supplement: S1 Table — The original, English, version of the questionnaire used in this study to assess reported health seeking behavior in eight villages in Kenema, Sierra Leone. (DOCX) [file pntd.0009576.s001.docx]

Supplemental information

**S1 Table. Questionnaire in English**

KENEMA, SIERRA LEONE

English

Health Seeking Behavior Questionnaire

| # | Question | Optional Answers | [for analysis: don’t fill in] |
| --- | --- | --- | --- |
| 1 | How old are you? |  | *Write age in complete years* |
| 2 | What is your sex? | Male Female | 1 2  *If male skip questions 6 & 7* |
| 3 | What is your Lassa history? | Never had lassa  Suspected Case (neg)  Confirmed Case (pos) | 1  2  3 |
| 4 | What is your religion? | Muslim  Christian  Other: | 1  2  3 |
| 5 | What is the highest level of education you have achieved? | No education  Primary  Secondary  Tertiary | 1  2  3  4 |
| 6a | If F:  Are you currently pregnant, or have been in the past 8 years? | Currently Pregnant  Not Pregnant, had child in past 8 years  Not pregnant, no child in past 8 years | 1  2  3 |
| 6b | How many kids do you have? |  |  |
| 6c | How old are your kids? |  |  |
| 6d | For each kid, where did you give birth? |  |  |
| 7a | If F: Where do you go for prenatal visits? | Government Hospital  Private Hospital  Traditional Birth Attendant  Home  Other: | 1  2  3  4  5 |
| 7b | If F: Where do you go to give birth? | Government Hospital  Private Hospital  Traditional Birth Attendant  Home  Other | 1  2  3  4  5 |
| 7c | If F: Where do you go for post birth follow up/immunizations? | Government Hospital  Private Hospital  Traditional Birth Attendant  Home  Other:_____ | 1  2  3  4  5 |
| 8 | When you seek health care where do you most often go? | Government Hospital  Private Hospital  Traditional Healer  Drug Shop  Self treatment  Other: _______ | 1  2  3  4  5  6 |
| 9 | What usually prompts you to seek health care? | Fever  Diarrhea  Headache/Nausea  Cough  Vomiting  Bleeding  Pain  Other: _______ | 1  2  3  4  5  6  7  8 |
| 10 | How long after symptoms do you usually seek health care? | Within 24 hours  Within 2-3 days  More than 3 days | 1  2  3 |
| 11 | What health issues prompt your community to seek care for you? | Headache/Nausea  Fever  Vomiting  Bleeding  Pain  Diarrhea  Cough  Other:_______ | 1  2  3  4  5  6  7  8 |
| 12 | What are the main barriers that prevent your community from seeking medical care from a health facility? | Distance  Cost  Time  Lack of sufficient information  Other: _____ | 1  2  3  4  5 |
| 13 | Do you usually get all the services you may need at the health facility? | Yes  No | 1  2 |
| 14 | Since the 2014 Ebola epidemic, some people say they have felt scared going to the hospitals. Others feel it is safer because more effort is being put into improving the health care system. Do you think it is any different? | Safer  Less safe  The same | 1  2  3 |
| 15 | Do you think your attendance at Kenema hospital has changed after the 2014 Ebola epidemic? | Yes, decreased attendance  Yes, increased attendance  No, similar attendance | 1  2  3 |
