# Supplementary material for: Health seeking behavior after the 2013–16 Ebola epidemic: Lassa fever as a metric of persistent changes in Kenema District, Sierra Leone
Source: PLoS Negl Trop Dis. 2021 Jul 14;15(7):e0009576. doi: 10.1371/journal.pntd.0009576 (PMC8312964; doi:10.1371/journal.pntd.0009576)
Supplement: S2 Table — The translated, Krio, version of the questionnaire used in this study to assess reported health seeking behavior in eight villages in Kenema, Sierra Leone. (DOCX) [file pntd.0009576.s002.docx]

Supplemental information

**S2 Table. Questionnaire in Krio.**

KENEMA, SIERRA LEONE

Krio

Health Seeking Behavior Questionnaire

| # | Question | Optional Answers | [for analysis: don’t fill in] |
| --- | --- | --- | --- |
| 1 | How old are you? |  | *Write age in complete years* |
| 2 | What is your sex? | Male Female | 1 2  *If male skip questions 6 & 7* |
| 3 | you be donee get lassa before ? | No get lassa  Suspected Case  Confirmed Case | 1  2  3 |
| 4 | What is your religion? | Muslim  Christian  Other: | 1  2  3 |
| 5 | ooh sai you tap na school? | No education  Primary  Secondary  Tertiary | 1  2  3  4 |
| 6a | If F: you be donee get bele before? | you get bele now  Not de pa bele now  You don’t get pickie past eight years | 1  2  3 |
| 6b | If F and have had kids in past 8 years: How many pickie you get ? |  |  |
| 6c | Whatena den age |  |  |
| 6d | For each kid-- Ooh sai you born den |  |  |
| 7a | Ooh sai you can go for clinic | Kenema Hospital  Private Hospital  Traditional Birth Attendant  Home  Other: | 1  2  3  4  5 |
| 7b | Ooh sai you can go for born | Kenema Hospital  Private Hospital  Traditional Birth Attendant  Home  Other | 1  2  3  4  5 |
| 7c | After where you born ooh sai you can go for checkup | Kenema Hospital  Private Hospital  Traditional Birth Attendant  Home  Other:_____ | 1  2  3  4  5 |
| 8 | Ooh sai you kin go where you seek | Kenema Hospital  Private Hospital  Traditional Healer  Drug Shop  Self treatment  Other: _______ | 1  2  3  4  5  6 |
| 9 | What tin kin make you go na hospital | Warm body  beleron  Headache/Nausea  Cough  Vomiting  Bleeding  Pain  Other: _______ | 1  2  3  4  5  6  7  8 |
| 10 | Wae you seek how long you kin take for go na hospital | one day  two days  More than 3 days | 1  2  3 |
| 11 | Which kin seek wae go make you people den kin take you na hospital | Headache/Nausea  warm body  Vomiting  Bleeding  Pain  belaron  Cough  Other:_______ | 1  2  3  4  5  6  7  8 |
| 12 | watin kin make people den nor kin go na hospital | Distance  Cost  Time  wae de not get better information  Other: _____ | 1  2  3  4  5 |
| 13 | You kin get all watin you kin go for na hospital | Yes  No | 1  2 |
| 14 | After ebola some people de fraid for go na hospital. Other people feel safe for go na hospital ibete. | bete for go  I no bete  anyone | 1  2  3 |
| 15 | When ebola don pas you hospital goan don change | less  more  the same | 1  2  3 |
