# Supplementary material for: Health seeking behavior after the 2013–16 Ebola epidemic: Lassa fever as a metric of persistent changes in Kenema District, Sierra Leone
Source: PLoS Negl Trop Dis. 2021 Jul 14;15(7):e0009576. doi: 10.1371/journal.pntd.0009576 (PMC8312964; doi:10.1371/journal.pntd.0009576)
Supplement: S3 Table — Data collected from the Viral Hemorrhagic Fever Consortium database. (DOCX) [file pntd.0009576.s003.docx]

Supplemental information

**S3 Table. Suspected Cases of Lassa Fever seen at Kenema Government Hospital, 2011-2019.**

| **Characteristic** | **2011**  **(n=828)** | **2012**  **(n=744)** | **2013**  **(n=523)** | **2014**  **(n=606)** | **2015**  **(n=429)** | **2016**  **(n=384)** | **2017**  **(n=277)** | **2018**  **(n=290)** | **2019**  **(n=351)** |
| --- | --- | --- | --- | --- | --- | --- | --- | --- | --- |
| **Gender** |  |  |  |  |  |  |  |  |  |
| **Female** | 494 (60.10) | 429 (59.01) | 293 (56.13) | 203 (53.99) | 218 (53.69) | 226 (58.85) | 155 (56.16) | 160 (55.36) | 172 (49.86) |
| **Male** | 328 (39.90) | 298 (40.99) | 229 (43.87) | 173 (46.01) | 188 (46.31) | 158 (41.15) | 121 (43.84) | 129 (44.64) | 179 (50.14) |
| **Age, years** |  |  |  |  |  |  |  |  |  |
| **<5** | 112 (14.36) | 104 (14.48) | 51 (9.79) | 24 (7.55) | 22 (5.57) | 79 (21.07) | 27 (9.82) | 35 (12.20) | 57 (16.29) |
| **5-19** | 190 (24.36) | 160 (22.28) | 123 (23.61) | 62 (19.50) | 72 (18.23) | 85 (22.67) | 55 (20.00) | 59 (20.56) | 37 (10.57) |
| **20-34** | 264 (33.85) | 237 (33.01) | 183 (35.12) | 92 (28.93) | 137 (34.68) | 113 (30.13) | 120 (43.64) | 99 (34.49) | 134 (38.29) |
| **35+** | 214 (27.44) | 217 (30.22) | 164 (31.48) | 140 (44.03) | 164 (41.52) | 98 (26.13) | 73 (26.55) | 94 (32.75) | 122 (34.86) |
| **Survival outcome** |  |  |  |  |  |  |  |  |  |
| **Discharged** | 73 (46.50) | 91 (61.49) | 90 (64.29) | 50 (22.52) | 15 (13.04) | 30 (53.57) | 47 (78.33) | 19 (46.34) | 8 (57.14) |
| **Died** | 84 (53.50) | 57 (38.51) | 50 (35.71) | 172 (77.48) | 100 (86.96) | 26 (46.43) | 13 (21.67) | 22 (53.66) | 6 (42.86) |
