# Supplementary material for: Health seeking behavior after the 2013–16 Ebola epidemic: Lassa fever as a metric of persistent changes in Kenema District, Sierra Leone
Source: PLoS Negl Trop Dis. 2021 Jul 14;15(7):e0009576. doi: 10.1371/journal.pntd.0009576 (PMC8312964; doi:10.1371/journal.pntd.0009576)
Supplement: S4 Table — Questionnaire respondent characteristics, including gender, religion and educational status. (DOCX) [file pntd.0009576.s004.docx]

Supplemental information

**S4 Table. Characteristics of Health Seeking Behavior Questionnaire Respondents.**

| **Question** | **N (%)** |
| --- | --- |
| **Gender** |  |
| Female | 129 (66) |
| Male | 65 (34) |
| **Lassa fever status** |  |
| Never had Lassa fever | 189 (98) |
| Lassa fever survivor | 4 (2) |
| **Religion** |  |
| Muslim | 173 (89) |
| Christian | 21 (11) |
| **Education level** |  |
| No education | 130 (68) |
| Primary education | 19 (10) |
| Secondary | 38 (20) |
| Tertiary | 3 (2) |
| **Where do you go for prenatal visits?** |  |
| Government hospital | 98 (87) |
| Private hospital | 6 (5) |
| Traditional birth attendant | 8 (7) |
| Home | 3 (4) |
| **Pregnancy status** |  |
| Currently pregnant | 19 (16) |
| Had child in past 8 years | 65 (54) |
| Did not have child in past 8 years | 43 (36) |
| **Where do you go to give birth?** |  |
| Government hospital | 100 (88) |
| Private hospital | 3 (3) |
| Traditional birth attendant | 8 (7) |
| Home | 5 (4) |
| **Where do you go for postnatal visits?** |  |
| Government hospital | 105 (95) |
| Private hospital | 6 (5) |
| Traditional birth attendant | 2 (2) |
| Home | 1 (1) |

Number of respondents for gender = 194; Lassa fever status = 193; religion = 194; education level = 190; place of prenatal visits = 113; pregnancy status = 121; place of delivery = 114; place of postnatal visits = 111. Response options for pregnancy status, place of prenatal visits, place of delivery, and place of postnatal visits were not mutually exclusive and subjects were permitted to select more than one response. As such, the sum of the percentages may exceed 100.
