# Supplementary material for: Health seeking behavior after the 2013–16 Ebola epidemic: Lassa fever as a metric of persistent changes in Kenema District, Sierra Leone
Source: PLoS Negl Trop Dis. 2021 Jul 14;15(7):e0009576. doi: 10.1371/journal.pntd.0009576 (PMC8312964; doi:10.1371/journal.pntd.0009576)
Supplement: S5 Table — Questionnaire responses for health seeking behavior pertinent questions. (DOCX) [file pntd.0009576.s005.docx]

Supplemental information

**S5 Table. Health Seeking Behavior.**

| **Question** | **N (%)** |
| --- | --- |
| **Where do you go most often to seek care?** |  |
| Government Hospital | 173 (91) |
| Private hospital | 20 (11) |
| Traditional healer | 2 (1.0) |
| Drug shop | 5 (3) |
| Self-treatment | 1 (1) |
| **What usually prompts you to seek health care?** |  |
| Fever | 150 (77) |
| Diarrhea | 24 (12) |
| Headache/nausea | 25 (13) |
| Cough | 10 (5) |
| Vomiting | 8 (4) |
| Bleeding | 1 (0) |
| Pain | 50 (26) |
| Other | 10 (5) |
| **When do you usually seek care?** |  |
| Within 24 hours | 114 (60) |
| Within 2-3 days | 52 (27) |
| More than 3 days | 24 (13) |
| **What health issues prompt your community to seek care for you?** |  |
| Fever | 128 (67) |
| Diarrhea | 19 (10) |
| Headache/nausea | 44 (23) |
| Cough | 5 (3) |
| Vomiting | 28 (15) |
| Bleeding | 3 (2) |
| Pain | 31 (16) |
| Other | 3 (2) |
| **What is the main barrier that prevents your community from seeking health care?** |  |
| Distance | 11 (6) |
| Cost | 163 (87) |
| Time | 8 (4) |
| Lack of sufficient information | 7 (4) |
| **Do you usually get all the services you may need at the health facility?** |  |
| Yes | 120 (68) |
| No | 56 (32) |
| **Do you think your attendance at Kenema hospital has changed after the 2014 Ebola epidemic?** |  |
| Decreased attendance | 36 (19) |
| Increased attendance | 135 (72) |
| Similar attendance | 16 (9) |
| **Since the 2014 Ebola epidemic, some people say they have felt scared going to the hospitals. Others feel it is safer because more effort is being put into improving the health care system. Do you think it is any different?** |  |
| Safer | 145 (78) |
| Less safe | 9 (5) |
| The same | 31 (17) |

Number of respondents for place sought for health care = 191; reason prompting health care = 194; time seeking health care = 190; issues prompting health care = 191; barriers for seeking health care = 187; services needed at health facility = 176; change in attendance following Ebola epidemic = 187; perceived difference in safety of hospital systems = 185 . Response options for issues prompting health care and barriers for seeking health care were not mutually exclusive and subjects were permitted to select more than one response. As such, the sum of the percentages may exceed 100.
