# Supplementary material for: Health seeking behavior after the 2013–16 Ebola epidemic: Lassa fever as a metric of persistent changes in Kenema District, Sierra Leone
Source: PLoS Negl Trop Dis. 2021 Jul 14;15(7):e0009576. doi: 10.1371/journal.pntd.0009576 (PMC8312964; doi:10.1371/journal.pntd.0009576)
Supplement: S6 Table — Demographic responses to the questionnaire broken down by village of residence. Eight villages in Kenema District are included. (DOCX) [file pntd.0009576.s006.docx]

Supplemental information

**S6 Table. Questionnaire demographic responses by village of residence.**

| **Characteristic** | **Village of residence** | | | | | | | | P value^1^ |
| --- | --- | --- | --- | --- | --- | --- | --- | --- | --- |
|  | 1  (n= 33) | 2  (n=23) | 3  (n=17) | 4  (n=18) | 5  (n= 20) | 6  (n= 20) | 7  (n= 33) | 8  (n= 30) |  |
| Population, N | 1,500 | 700 | 2,000 | 800 | 600 | 2,100 | 3,000 | 1,500 | <.001 |
| Distance to KGH (km) | 51.5 | 46.7 | 22.5 | 27.4 | 33.8 | 38.6 | 38.6 | 41.8 | --- |
| Gender, n (%) |  |  |  |  |  |  |  |  |  |
| Female | 23 (70) | 14 (61) | 12 (71) | 12 (67) | 14 (70) | 9 (45) | 21 (64) | 24 (80) | .399 |
| Male | 10 (30) | 9 (39) | 5 (29) | 6 (33) | 6 (30) | 11 (55) | 12 (34) | 6 (20) |  |
| Age, mean (STD) | 37 (1.1) | 38 (1.3) | 35 (0.9) | 43(1.3) | 34 (1.4) | 41 (1.1) | 37 (1.3) | 40 (1.4) | .028 |
| Religion, n (%) |  |  |  |  |  |  |  |  |  |
| Muslim | 33 (100) | 20 (87) | 16 (94) | 17 (94) | 18 (85) | 20 (100) | 23 (70) | 26 (87) | .004 |
| Christian | 0 (0) | 3 (13) | 1 (6) | 1 (6) | 2 (15) | 0 (0) | 10 (30) | 4 (13) |  |
| Education, n (%) |  |  |  |  |  |  |  |  |  |
| Some | 1 (3) | 1 (6) | 1 (8) | 2 (14) | 2 (15) | 2 (12) | 5 (23) | 5 (21) | .449 |
| None | 28 (97) | 17 (94) | 11 (92) | 12 (86) | 11 (85) | 15 (88) | 17 (77) | 19 (79) |  |

STD = Standard deviation. --- = fixed data and p values were not calculated. Differences between overall time period frequencies and characteristic frequencies are due to missing characteristic data.

^1^P values comparing populations were considered as the proportion sampled per population. Comparisons for categorical and continuous responses were calculated using Fisher’s Exact Test and the Wilcoxon Test, respectively.
