# Supplementary material for: Health seeking behavior after the 2013–16 Ebola epidemic: Lassa fever as a metric of persistent changes in Kenema District, Sierra Leone
Source: PLoS Negl Trop Dis. 2021 Jul 14;15(7):e0009576. doi: 10.1371/journal.pntd.0009576 (PMC8312964; doi:10.1371/journal.pntd.0009576)
Supplement: S7 Table — Results from a logistic regression analysis assessing satisfaction with health services in Kenema District. (DOCX) [file pntd.0009576.s007.docx]

Supplemental information

**S7 Table. Logistic regression assessing satisfaction with health services in 2018 in Kenema district.**^1^

| **Characteristic** | **OR (95% CI)** | **P value** |
| --- | --- | --- |
| **Age** (with reference to 18 to 27 years) |  |  |
| 28-36 | 0.96 (0.31, 2.96) | .293 |
| 37-45 | 1.49 (0.43, 5.17) | .059 |
| 45+ | 0.87 (0.26, 2.92) | .487 |
| **Sex** (with reference to female) | 2.10 (0.87, 5.10) | .101 |
| **Religion** (with reference to Christian) | 1.21 (0.30, 3.87) | .789 |
| **Education** |  |  |
| Primary | 0.92 (0.23, 3.69) | .723 |
| Secondary/Tertiary | 0.52 (0.19, 1.41) | .265 |
| **Village** (with reference to Village 1) |  |  |
| 2 | 1.34 (0.25, 7.24) | .099 |
| 3 | 0.76 (0.14, 4.20) | .516 |
| 4 | 0.08 (0.02, 0.40) | .001 |
| 5 | 0.62 (0.11, 3.37) | .725 |
| 6 | 0.14 (0.03, 0.57) | .006 |
| 7 | 0.94 (0.20, 4.41) | .216 |
| 8 | 0.59 (0.14, 2.57) | .730 |

^1^Predictor variables include age, sex, religion and education analyzed among 173 observations.
