# Supplementary material for: Health seeking behavior after the 2013–16 Ebola epidemic: Lassa fever as a metric of persistent changes in Kenema District, Sierra Leone
Source: PLoS Negl Trop Dis. 2021 Jul 14;15(7):e0009576. doi: 10.1371/journal.pntd.0009576 (PMC8312964; doi:10.1371/journal.pntd.0009576)
Supplement: S8 Table — Results from a logistic regression analysis assessing perceived hospital safety in 2018 in Kenema District. (DOCX) [file pntd.0009576.s008.docx]

Supplemental information

**S8 Table. Logistic regression results for perceived hospital safety in 2018 in Kenema district**

| **Characteristic** | **OR (95% CI)** | **P value** |
| --- | --- | --- |
| **Age** |  |  |
| 28-36 | 1.58 (0.52, 4.83) | .648 |
| 37-45 | 3.99 (0.92, 17.22) | .042 |
| 45+ | 0.88 (0.28, 2.76) | .315 |
| **Sex (**with reference to female) | 2.10 (0.82, 5.34) | .124 |
| **Religion** (with reference to Christian) | 0.64 (0.14, 2.91) | .565 |
| **Education** |  |  |
| Primary | .961 (0.24, 3.88) | .575 |
| Secondary/Tertiary | 2.06 (0.62, 6.83) | .247 |
| **Village** |  |  |
| 2 | 2.33 (0.51, 10.65) | .975 |
| 3 | --- | .958 |
| 4 | 1.43 (0.33, 6.27) | .962 |
| 5 | 0.88 (0.21, 3.80) | .948 |
| 6 | 0.96 (0.25, 3.73) | .951 |
| 7 | 1.45 (0.36, 5.88) | .962 |
| 8 | 1.18 (0.32, 4.36) | .957 |

Predictor variables age, sex, religion, education, and village analyzed among 166 individuals. --- = same responses between comparison groups and insufficient variability computing confidence interval.
