# Supplementary material for: Health seeking behavior after the 2013–16 Ebola epidemic: Lassa fever as a metric of persistent changes in Kenema District, Sierra Leone
Source: PLoS Negl Trop Dis. 2021 Jul 14;15(7):e0009576. doi: 10.1371/journal.pntd.0009576 (PMC8312964; doi:10.1371/journal.pntd.0009576)
Supplement: Attachment — Submitted filename: PTND_D_20_01152_ResponseToReviewers_Koch.docx [file pntd.0009576.s009.docx]

**POINT-BY-POINT REVIEWER RESPONSES**

Reviewer 1:

*Dear Reviewer 1,*

*We truly thank you for your careful review of our manuscript and constructive comments and have used this feedback to significantly revise (and, we believe substantially improve) the manuscript. Please find below our point-by-point italicized responses (including line numbers referencing the modification changes in the revised manuscript) to your critiques and comments. We do not believe that any of the changes in the revised manuscript altered the general conclusion or results presented in the original manuscript.*

Results

**1.1.** In general, the results are clearly written and easy to follow, although I do feel as if several of the figures could be displayed in different ways. For example, given that all the panels of Figure 4 could be represented as pie charts to more clearly represented the proportions (although this is just the reviewer's preference, they are fine as they are!).

*We agree with the reviewer and have changed the panels in Figure 4 to pie charts. [Figure 4, Line 473]*

General Comments

**1.2.** Statistical strength of the data is mentioned numerous times in the manuscript, with references to the relevant figures. However, there is no visual representation of statistics on the figures which would help the reader quickly understand the differences between data groups. If the authors could find a way to add representations of significance to the figures I think this would improve the manuscripts readability.

*We thank the reviewer for this observation and have added significance values to Figure 3B.*

Specific Comments:
**1.3.** I think it would be useful for the reader to have a map of the area sampled, so the reader could have an understanding of where villages 1-7 are located both in relation to Kenema and in relation to Sierra Leone as a whole.

*We appreciate this comment from the reviewer. While we agree that this would help the reader understand where the villages are in relation to Kenema, we have chosen to not include a map for reasons of confidentiality.*

**1.4.** Table 1: I'm unclear as to what line the p values are referring to? There seems to be one value per characteristic, although each characteristic has multiple criteria?

*We thank the reviewer for this observation and have revised the note for Table 1as ^“^Fisher’s Exact and Kruskal-Wallis tests used to test for general differences between pre-and post-Ebola time periods for categorical and continuous characteristics, respectively.”*

**1.5.** Line 374: Should be villages 4 and 6?

*We thank the reviewer for this observation and have made the necessary changes in the revised manuscript. (Line 540)*

Reviewer 2:

*Dear Reviewer 2,*

*We truly thank you for your careful review of our manuscript and constructive comments and have used this feedback to significantly revise (and, we believe substantially improve) the manuscript. Please find below our point-by-point italicized responses (including line numbers referencing the modification changes in the revised manuscript) to your critiques and comments. We do not believe that any of the changes in the revised manuscript altered the general conclusion or results presented in the original manuscript.*

General Comments

**2.1.** Line 390: "series" is not clear and should be reworded.

*We agree with the reviewer and have revised the manuscript accordingly. The sentence now reads: “The number of patients evaluated in 2013 for potential Lassa fever was consistent with this trend*.” *(Lines 558-559)*

**2.2.** Lines 490-494: These sentences should be revised for more clarity.

*We thank the reviewer for this observation and have made the suggested changes in the revised manuscript. Specifically, the sentences in question have been replaced as “If, in line with our findings in this paper, HSB decreased during the COVID pandemic and remains below baseline following the pandemic, such information will be vital for appropriate mitigation efforts. Particularly in lower resourced health systems, already confronting a sustained impact on health seeking behavior from the West African Ebola epidemic, monitoring how that may be further impacted by COVID-19 will be critical.” (Lines 764-768)*

Reviewer 3:

*Dear Reviewer 3,*

*We truly thank you for your careful review of our manuscript and constructive comments and have used this feedback to significantly revise (and, we believe substantially improve) the manuscript. Please find below our point-by-point italicized responses (including line numbers referencing the modification changes in the revised manuscript) to your critiques and comments. We do not believe that any of the changes in the revised manuscript altered the general conclusion or results presented in the original manuscript.*

Methods

**3.1.** Line 188 - "rates was," - I believe the comma should be placed after "rates" instead of "was".

*We agree with the reviewer and have made these suggested changes in the revised manuscript. (Line 272)*

**3.2.** Line 194 - "VHFC" - This abbreviation is used here without the full term having appeared in the text. Please include this prior to the abbreviation.

*We agree with the reviewer and have made this suggested change in the revised manuscript. (Lines 277-278)*

**3.3.** Lines 202-204 - "Fisher's Exact and Kruskal-Wallis tests were used to compare categorical and continuous characteristics, respectively, between pre-and post-Ebola time periods. Significance tests were performed using the SAS statistical application." - It would aid the reader in interpreting the results if the authors could provide more details of the significance tests performed. For example, the meaning of a statistically significant p-value in the specific context of the two tests used.

*We thank the reviewer for this observation and have made the suggested changes in the revised manuscript. Specifically, these sentences were added: “Type 1 error threshold was set at 5%, and those p-values less than this threshold were considered as statistically significant. Statistical significance focused on differences observed between pre- and post-Ebola time periods (2011-13 and 2017-19, respectively) that were not due to chance alone.” (Lines 289-292)*

**3.4.** Lines 206-207 - "HSB responses were analyzed to identify potential predictors of responses to the Lassa pertinent questions" - Does this statement mean HSB responses were analysed to determine predictors for the specific responses to the Lassa fever related questions? Please reword to improve clarity of meaning.

*We thank the reviewer for this observation. The sentences in question have been reworded for increased clarity in the revised manuscript. Specifically, the sentence was reworded as: “General HSB questionnaire responses (questions 1, 2, & 4-12) were analyzed to identify potential predictors of responses to the specific study pertinent questions (3 & 13-15).” (Lines 299-300)*

**3.5.**  Line 207 - "The analysis was performed by running logistic regression models in STATA" - Could you please provide more specific details of your logistic regression models, including the selection process of variables used in the final model?

*We thank the reviewer for this observation and have added in the following sentence to the revised manuscript. “The logistic regression analyses were performed using full main effects (no interaction) models, where outcomes were dichotomized and modeled against subject age, gender, religion, education, and village of residence.”(Lines 302-304)*

Results

**3.6.**    Line 214 - "In the three-year period (2017-19) following the West African Ebola outbreak, there was a decline..." - Please revise this wording because it currently implies a decline over the period of 2017-2019, rather than compared to the previous 6 years. Fig1A shows a slight increase from 2017 to 2019.

*We agree with the reviewer and have revised the statement as follows: “In the three-year period (2017-19) following the West African Ebola epidemic, as compared to the six years prior to the epidemic, there was a decline in the numbers of subjects presenting to the Kenema Government Hospital (KGH) Lassa ward with suspected Lassa fever and the numbers of patients admitted to the KGH Lassa ward (Fig 1, S3 Table).” (Lines 313-316)*

**3.7.**      Line 222 - "(p = .034, Table 1)" - The p-value here is inconsistent with the value in Table 1 (0.049). Please review and remove any inconsistencies.

*We thank the reviewer for this observation and have removed this inconsistency in the revised manuscript. (Line 327)*

**3.8.**        Line 259 - "However, in 2019 more males than females presented to KGH." - It would be more informative to the reader if the value difference between female and male presentations was provided here.

*We thank the reviewer for this observation and have added revised the statement as follows: “However, in 2019 more males (179) than females (172) presented to KGH.” (Line 372)*

**3.9.**      Line 261 - "(p = .029; Table 1)" - The p-value here is inconsistent with the value in Table 1 (0.005). Please review and remove any inconsistencies.

*We thank the reviewer for this observation and have removed the inconsistency in the revised manuscript. (Line 373)*

**3.10.**       Lines 275-276 - "The decline in suspected cases remained statistically significant across all age groups (Fig 2)." - Please consider revising this sentence to improve clarity of meaning. For example, it should be made explicit if this is comparing the three years pre-Ebola to the three years post-Ebola. Furthermore, Figure 2 does not present any data regarding the statistical significance of the change in number of suspected cases per age group, therefore I suggest the authors either remove this claim of statistical significance or substantiate it via other evidence presented in the paper.

*We thank the reviewer for this observation and have removed this claim in the revised manuscript.*

**3.11.**      Lines 276-277 and lines 282-283 - "There were no major changes in the proportion of suspected Lassa fever cases in most age groups pre- vs post- epidemic" and "The differences pre-epidemic and post-epidemic in the age group proportions among suspected Lassa fever cases was significant" - These two claims are contradictory to each other. Please revise the text to remove this inconsistency and more clearly state the results in the table.

*We thank the reviewer for this observation and have added revisions to the manuscript. The lines now read as follows: “There were no major changes in the proportion of all annual suspected cases of Lassa fever associated with each age group, pre- versus post-epidemic (Fig 2B, Table 1)” and “The decline in the total number of suspected Lassa fever cases pre-epidemic versus post-epidemic among each age group was significant (p < .001, Table 1)” respectively. (Lines 389-390, and lines 401-432)*

**3.12.**      Line 273 - Figure 2B - The legend for age groups shows the category of 4-19 years, but the text specifies the age range of 5-19 years. Please revise this as appropriate.

*We agree with the reviewer and have made the suggested changes in the revised manuscript. The legend now reads 5-19 years. (Figure 2B)*

**3.13.**        Lines 277-278 - "The 20-24 year old age group was the largest proportion..." - I believe this should be the "20-34" year old age group. Furthermore, I would recommend rewording slightly to say that "The 20-34-year-old age group was the age group with the largest proportion..."

*We agree with the reviewer and have made the suggested changes in the revised manuscript. (Line 397)*

**3.14.**      Line 280 - "(35%, Fig 2B, Table 1)" - The proportion for 20-34 in Table 1 is recorded as 37%. Please review and correct as appropriate to remove any inconsistency.

*We thank the reviewer for this observation and have removed this inconsistency in the revised manuscript. (Line 399)*

**3.15.**     Lines 280-281 - "...there was a reduction in the number of children and teenagers..." - Please consider rewording this sentence to make it explicit that it is the reduction in the proportion of suspected cases being referred to and not simply the raw number.

*We agree with the reviewer and have made the suggested changes in the revised manuscript. (Lines 400-401)*

**3.16.**    Line 286 - "The suspected case fatality rate..." - It may improve the readability of this sentence to rephrase this as "The case fatality rate of suspected cases...". Additionally, subsequent uses of "suspected CFR" should be similarly reworded or simply be replaced with "CFR".

*We agree with the reviewer and have made the suggested changes in the revised manuscript. (Line 406)*

**3.17.**      Lines 287-289 - "the West African Ebola epidemic" and "the Ebola outbreak" - Please consider rewording to be consistent in term use. Throughout the text, the latter of these appears to be more frequently used.

*We thank the reviewer for this observation, and have chosen to use the more formal term West African Ebola epidemic throughout the revised manuscript.*

**3.18.**      Lines 292-294 - "Over the time-period studied, the numbers of deaths and discharges amongst cases admitted to the Lassa Ward consistently declined in parallel to the numbers of overall suspected cases." - It is unclear what is meant by "consistently declined in parallel", when the numbers presented in Figure 3A are based on the same numbers as Figure 1B. Is this stating that the three-year-average of both discharges and deaths has fallen between the pre-Ebola period and the post-Ebola period in the same way that the three-year average fell between these two periods for number of admitted cases? Please reword this sentence to improve clarity of meaning.

*We agree with the reviewer and have added revisions to the manuscript. The line now read as follows: “Over the time-period studied, the numbers of deaths and discharges amongst cases admitted to the Lassa Ward declined between the pre-Ebola and post-Ebola periods in parallel with the decline in number of admitted cases.” (Lines 412-414)*

**3.19.**    Line 307 - Figure 3B - This figure appears to be incomplete. It only shows a single line with red markers for values, but it is described as the fatality rate "by age category". Please revise such that this figure shows the data for all age categories.

*We thank the reviewer for this observation. This figure is not reflecting fatality rate by age, rather overall fatality rate by year. The reference to age categories has been removed from the figure legend. (Figure 3B)*

**3.20.**       Lines 311-312 - "Of those completing the questionnaire, 89% were Muslim, 66% were female, and 69% had no education." - Please consider revising this summary of results such that each category of results is provided in its own context, e.g. Of the religions specified by the respondents, 89% were Muslim, followed by...

*We thank the reviewer for this observation and revised the sentences accordingly. The sentences now read as follows: “Of the religions specified by respondents, 89% were Muslim, followed by 11% that were Christian. Of the genders specified by respondents, 66% were female and 34% were male. Of the education levels specified by respondents, 68% received no education, followed by 10% receiving primary education, 20% receiving a secondary education, and 2% receiving a tertiary education (S4 Table).” (Lines 446-449)*

**3.21.**      Line 313 - "The majority of women reported having two living children." - Please include the percentage value for this majority. Furthermore, the term "living children" is ambiguous. Please consider rewording to be more precise.

*We thank the reviewer for this observation. The percentage of women reporting having two living children has been updated in the revised manuscript. Further, a footnote has been added to clarify the ambiguous term ‘ living children.’ The footnote reads as follows: ^“^Culturally, given many female respondents had lost children, it was accepted to refer to children that were still alive, whereas medically, any full-term pregnancy may have been relevant.”(Line 450)*

**3.22.**      Lines 313-315 - "88% of women..." - Please spell out the percent value here as sentences should not start with a number value.

*We agree with the reviewer and have made the suggested changes in the revised manuscript.(Line 451)*

**3.23.**      Lines 317-318 - "When seeking health care, 89% of respondents reported that they go to a government hospital" - This value is reported as 90% in S5 Table. Please review and correct the inconsistency as appropriate.

*We thank the reviewer for this observation and have added the relevant information to the S5 table in the revised manuscript. (Lines 463-464)*

**3.24.**      Lines 318-319 - "Only 4% reported they used a form of self-treatment or a traditional healer" - The values in S5 Table for self-treatment (0%) and traditional healer (1.0%) do not add up to 4%. Please review and correct the inconsistency as appropriate.

*We thank the reviewer for this observation and have removed this inconsistency in the revised manuscript. (Lines 464-465)*

**3.25.**        Lines 319-320 - "68% of respondents confirmed receiving all their needed services at their health facility" - While shown in Figure 4B, this information is not presented in S5 Table. Please ensure that all survey answers are presented in the corresponding tables in the supplementary text.

*We thank the reviewer for this observation and have added the relevant information to the S5 table in the revised manuscript.*

**3.26.**      Lines 320-321 - "individuals reported they were likely to seek care 24 hours after symptoms arose..." - According to the survey questions listed in the supplementary text, this should be "within 24 hours".

*We agree with the reviewer and have made the suggested changes in the revised manuscript. (Line 467)*

**3.27.**       Lines 321-322 - "As noted by 87% of respondents, cost was the most common barrier preventing subjects from seeking care at a health facility" - Please consider an alternative expression to "As noted by", such as "According to". Furthermore, please consider replacing "subjects" with "respondents" to increase consistency.

*We agree with the reviewer and have made the suggested changes in the revised manuscript. The sentence now reads as follows: “According to 87% of respondents, cost was the most common barrier to seeking care at a health facility (Fig 4D).” (Lines 467-468)*

**3.28.**       Lines 322-324 - "72% of respondents said their attendance at a healthcare facility had increased since the epidemic, and 19% said it had decreased" - While shown in Figure 4E, this information is not presented in S5 Table. Please ensure that all survey answers are presented in the corresponding tables in the supplementary text.

*We thank the reviewer for this observation and have added the relevant information to the S5 table in the revised manuscript.*

**3.29.**        Lines 324-325 - "78% of respondents stated... ...remained the same" - While shown in Figure 4F, this information is not presented in S5 Table. Please ensure that all survey answers are presented in the corresponding tables in the supplementary text.

*We thank the reviewer for this observation and have added the relevant information to the S5 table in the revised manuscript.*

**3.30.**       Lines 339-340 - "There were no significant differences in responses to health utilization or perceived safety of hospitals between men and women, or distance from KGH (S7 Table, S8 Table)." - S7 Table's label suggests it describes satisfaction with health services rather than utilization. Please revise to correct this inconsistency. Furthermore, these two tables only present OR and 95% CI. Please consider including the p-value to these tables to better substantiate claims of statistical significance.

*We thank the reviewer for this observation and have added p-values to tables S7 and S8 to substantiate significance claims.*

**3.31.**       Lines 340-341 - "With the exception of a group without a recorded age (group 5) respondents were likely to say they received all the services they needed at their health facility (S8 Table)." - This is the first use of a "group" in the text and serves no meaningful purpose. If this term is deemed necessary, please define it in the Methods section of the manuscript. Furthermore, S8 Table only lists four categories for age. Please review and revise as appropriate.

*We thank the reviewer for this observation and have removed the group for missing age values from the results.*

**3.32.**      Providing the p-values specific to these statements in the main text will also add to the reader's understanding of the results.

*We agree with the reviewer and have added adjusted odds ratios and confidence intervals into the revised manuscript to substantiate these statements. The sentence now reads: “Self-reported satisfaction for health services was significantly higher among males than females (adjusted odds ratio [aOR] = 2.1, 95% confidence interval [95% CI]= [0.87, 5.10]).” (Lines 493-494)*

**3.33.**     Lines 348-355 - "Symptoms triggering heath seeking behavior" - The interpretation of these results is over-simplifying the frequency of symptoms because it only demonstrates whether the occurrence of symptoms leads to HSB, but does not quantify the frequency of each symptom. For example, the low frequency recorded for bleeding may be more attributable to the survey participants simply not having experienced those symptoms rather than them not seeking health care for that specific symptom. Without quantitative data on frequency of symptoms, it is not possible to fully describe which symptoms are most and least likely to lead to HSB. Therefore, care should be taken to not overstate the meaning of the recorded responses. This lack of quantitative data should be discussed as one of the limitations of the study.

*We agree with, and thank, the reviewer for this observation. We made sure to specific, and clarify, in the revised manuscript that the data does not account for frequency of systems. The follow sentences have been added: “Responses to the questionnaire were used to identify how likely individuals are to initiate HSB for specific symptoms, both for themselves and their communities (Fig 5). The frequency of symptoms was not determined through the questionnaire and could explain varying levels of HSB by symptom.” (Lines 502-505)*

**3.34.**       Lines 365-377 - "Variation in health utilization and satisfaction among villages" - It is not clear why Village 1 was chosen as a reference village for this village level analysis. This should be clearly described in the Methods section.

*We thank the reviewer for this observation and have added rational for why Village 1 was chosen as a reference village in the revised manuscript. The follow sentences have been added: “Village 1 was selected to be the reference village as it was one of two villages with the most respondents. One of these two villages was arbitrarily chosen as the reference village.”(Lines 305-307)*

**3.35.**   Lines 374 - "...respondents in villages 3 and 6..." - based on Figure 6A I believe this should be villages 4 and 6. Please review and revise as appropriate.

*We agree with the reviewer and have made the suggested changes in the revised manuscript (Line 540)*

Conclusions

**3.36.**  Line 397 - "There is no evidence that the incidence of Lassa fever is decreasing in West Africa" - Please provide a reference immediately following this statement.

*We thank the reviewer for this observation and have rewritten the relevant sentences in the revised manuscript for clarity, and provided appropriate references. The sentences read as follows: “The evidence indicates that the incidence of Lassa fever may actually be increasing in West Africa (43). The largest outbreaks of Lassa fever in Nigeria have occurred in the past three years (44, 45). From 2017-2018, alone, Nigeria experienced their largest ever recorded LF outbreak, which was longer, and included more severe cases than previously seen (46). Additionally, LF has been detected with increasing frequency in neighboring countries Togo and Benin (47).”(Lines 566-571)*

**3.37.**  Line 399 - "...in Togo and Benin [12]" - Please check that this is the correct reference and replace if necessary.

*We thank the reviewer for this observation. Reference [12] was the incorrect reference for this statement and has been revised to reflect the proper source. (Line 571)*

**3.38.**  Line 413 - "one in five respondents still reporting not feeling safer in hospital care" - The group of respondents who specified "same" for their feeling of safety should not be included as possible cause for decline in HSB for Lassa fever. Please revise this statement to only include those who answered "less". Please also considering revising the statement on lines 450-451 similarly.

*We thank the reviewer for this observation. We have added the following sentences to the revised manuscript for increased clarity: “Given the documented decline in HSB during the epidemic, individuals who reported feeling similar safety levels at hospitals since the epidemic are still likely a reflection of a decline in HSB as compared to pre-epidemic levels. As such, for the purposes of this study, they are being considered within the category of individuals who are not reporting increased safety in hospital care. (Lines 576-608)*

**3.39.**  Lines 450-453 - "...around 20% of respondents... ...non_lassa visits." - The connection being drawn between a lack of increase in trust in healthcare facilities and a 20% decline in presentation for suspected Lassa fever at KGH is unclear. Please revise and elaborate on this discussion and further describe how the authors drew this connection from the analysis.

*We thank the reviewer for this observation. The sentences have been revised as follows. “While the majority of respondents reported hospitals as “safer after the epidemic,” and have “increased their attendance at health facilities since the epidemic,” around 20% of respondents did not report increased trust in healthcare facilities. Given that individuals do not present more than once with Lassa fever, this group of 20%, if extrapolated to the regional population, could account for the identified decline in hospital presentation for suspected Lassa fever.” (Lines 646-650)*

**3.40.**   Line 451 - "Given that individuals do not present more than once with Lassa fever" - Please provide a reference for this claim.

*We thank the reviewer for this observation. There is no citation for this, and reinfection rates are not well-understood. There have been anecdotally very few observations of repeats in the name/chiefdom/village/district combinations in the clinical database (but there have been a few). As such, we have removed this statement from the manuscript.*

**3.41.**   Lines 465 - "One can imagine... ...of Lassa fever." - Please revise this statement to increase clarity of meaning, in particular the meaning of "override a decreased concern".

*We thank the reviewer for this observation. The sentences have been revised as follows. “In conjunction with a potential decrease in concern for the early signs of Lassa fever, one can imagine a scenario in which any cost, which is the primary barrier to seeking care reported by 87% of the respondents, would be sufficient to override any inclination to seek medical attention.” (Lines 677-680)*

General Comments

**3.42.**     Throughout the paper, where percentage values are presented, I would recommend providing at least 2 decimal places of precision. Please ensure this is consistently applied to all data in both the main manuscript and the supplementary text.

*We agree with the reviewer and have made the suggested changes in the revised manuscript.*

**3.43.**       Abbreviated terms should be explicitly stated in full for their first use in each major section, i.e. Introduction, Methods, Results, Discussion. For example, HCW is defined as health care workers in the Introduction, but not in the Discussion section.

*We agree with the reviewer and have made the suggested changes in the revised manuscript.*

**3.44.**        A number of minor spelling and grammar mistakes exist through the paper, please proofread the entire manuscript to correct these.

*We thank the reviewer for this observation and have proofread the revised manuscript.*

**3.45.**        The Methods section does not provide sufficient details describing why the authors adopted the statistical approaches used in this study. Furthermore, the hypotheses being tested in this study are not made clear, which makes it difficult to accurately interpret the results.

*We thank the reviewer for this observation.*

a. *To address insufficient details regarding the statistical approaches adopted, the following sentences have been added: “Nonparametric approaches (Fisher’s Exact and Kruskal-Wallis tests) were used to account for departures in diagnostics for their analog parametric approaches (Pearson’s chi square and ordinary one-way analysis of variance). The logistic regression approaches were applied due to the categorical nature of the data captured in the HSB survey. (Lines 286-289)*
b. *“To increase clarity regarding the hypothesis being tested in this study, the following sentence has been added to the Introduction “Based on the incredible shock to the system (societal, healthcare, governmental, economic etc.) and clear evidence of a dramatic change in health seeking behavior during the epidemic across the nation, the authors hypothesized that there would remain an impact on hospital attendance for Lassa fever observed in the population years after the epidemic concluded.” (Lines 160-164)*

**3.46.**    The Results section contains incomplete figures, incorrect references to values in tables, and unclear descriptions of some of the results recorded.

*We thank the reviewer for this observation and have worked to reduce these inconsistencies.*

**3.47.**      The Discussion section presents a wide variety of topics related to HSB, however, there is very limited discussion specific to the findings of this study. Furthermore, there is no discussion of the results of the logistic regression analyses performed in the study. The discussion also comments that the individual village sample sizes were too small for inter-village comparison, however these comparisons are still presented in the results with no further elaboration in the Discussion section.

*We thank the reviewer for this observation and strove to incorporate these comments into the revised manuscript. The following sentence have been added to the discussion: “While questionnaire responses varied by village, the sample sizes were too small to identify which specific differences among villages might explain the overall observation. Based on the analysis completed, there were differences amongst villages in the proportion of respondents that felt they got all the services they needed at their health facility. However, this difference was not explained by gender, religious affiliation, or level of education. Distance from KGH, while considerable, did not explain the different responses. While others have reported differences in health seeking behavior among genders and age groups, our limited sample size was unable to identify an association.”(Lines 664-671)*

**3.48.**      S1 and S2 Tables - In both languages for the questionnaire, there is a note next to question 2 to skip to question 8 if the interviewee is male, which would skip over questions relating to Lassa fever history, education, and religion, but the numbers presented in S3 Table suggest that these questions were answered by both male and female participants. Could you please review the questionnaires included in the supplementary text to ensure they accurately represent the actual questionnaire performed based on the results presented.

*We thank the reviewer for this observation and have made the suggested changes in S1 and S2 tables in the revised manuscript.*

**3.49.**     S4 Table lists the numbers of respondents per question, but some of these are inconsistent. For example, level of education is listed as being answered by 160 participants in the note at the bottom of the table, but the numbers within the table sum to 191. Furthermore, the main text on lines 311-312 references the percentage value for no education of all who completed the questionnaire rather than of the 191 who answered this question, without any mention of the different questions of the survey being answered by varying numbers of participants.

*We thank the reviewer for this observation and have revised the manuscript accordingly.*

**3.50.**     S7 and S8 tables list the age categories 28-36, 37-45, 45+, and missing, whereas the main text uses the categories <5, 5-19, 20-34, and 35+, but there is no explanation for this difference in categorisation of ages. Please review this different categorization and provide an explanation in the main text when the age categories from these tables are first referenced.

*We thank the reviewer for this observation. We have added the following sentences to clarify the use of varying age categories: “**For this analysis, age categories, 18-27, 28-36, 37-45, and 45+, were selected to accommodate the age inclusion criteria for the HSB questionnaire.” (Lines 499-499)*

Abstract

**3.51.**     Line 39 - It is stated that "dramatic underreporting and substantial declines in hospital presentations", but the scale of this change is not clear. It would assist in understanding the magnitude of the change in hospital presentations if some metric were used to compare pre- and post-Ebola outbreak.

*We agree with the reviewer and have added additional detail in the revised manuscript. The revised sentence reads as follows: “Changes in health seeking behavior (HSB) during the outbreak resulted in dramatic underreporting and substantial declines in hospital presentations to public health facilities, resulting in an estimated tens of thousands of additional maternal, infant, and adult deaths per year.” (Lines 40-41)*

Author Summary

**3.52.**     Line 76 - "a questionnaire given to nearly 200 area residents" - The term "area residents" is ambiguous. Please consider alternate wording. Furthermore, the abstract states that 200 surveys were given but 194 (nearly 200) were completed. Please consider revising wording to more accurately describe the numbers presented.

*We agree with the reviewer and have revised the sentence as follows: “Responses to a questionnaire completed by 194 Sierra Leonean residents suggested that confidence in the healthcare system was not fully restored.”(Line 85)*

Introduction

**3.53.**       Line 96 - "While Ebola was directly killing thousands" - The expression "directly killing thousands" could be better reworded as "directly responsible for the deaths of thousands". Additionally, it may be helpful to provide more specific figures in this sentence (perhaps contained in the references), as the term "thousands" is used twice in the sentence, which does not provide a clear sense of relative scale.

*We agree with the reviewer and have revised the sentence as follows: “While Ebola was directly responsible for thousands of deaths (approx. 4000), changes in health seeking behavior (HSB) due to documented fear, misinformation, distrust, and a reduction in medical services, resulted in considerable non-Ebola mortality that continues to this day (10-13).”(lines 109-112)*

**3.54.**      Line 96 - "...changes in health seeking behavior (HSB) due to..." - Given that HSB is a key topic of this paper, it would be beneficial to the reader for the authors to provide their own definition of HSB. Furthermore, the Introduction later states that a goal of the study was to quantify HSB, therefore it would be beneficial to provide examples of how HSB can be quantified along with this definition of HSB.

*We thank the reviewer for this observation and have added the following sentences to the revised manuscript’s Introduction: “Health seeking behavior, as assessed in this study, is defined as “a sequence of remedial actions individuals undertake to rectify perceived ill health” (21). The underlying assumption is that health is influenced by behavior and behavior itself is subject to modification, as influenced by external and internal factors (22). Commonly used proxies for health seeking behavior have been healthcare consumption patterns, provider visits, and self-reported care seeking.”(Line 142-147)*

**3.54.**     Line 98 - "At the height of the epidemic, there was a 31- 37% drop in facility-based deliveries" - The references show that this sentence is referring to child delivery but manuscript text should be more explicit. Furthermore, it is not made clear how this directly relates to the previous statement regarding additional deaths due to changes in HSB. It may be beneficial to the reader to add some additional context such as what impact the changes in HSB have had on maternal health. Additionally, the geographical scope of this figure is unclear. Is the drop in deliveries that of Sierra Leone or a region therewithin?

*We thank the reviewer for this observation and the sentences have been revised as follows: “At the height of the epidemic, there was a 31- 37% drop in facility-based child deliveries and an 80% decrease in pediatric admissions for malaria in Sierra Leone (10, 15). Such reduction in healthcare provision was predicted to result in an additional 4,022 maternal deaths per year and 6,700 additional infant deaths per year in the region (10).” (Lines 112-116)*

**3.55.**     Lines 103-104 - "the attitudes, beliefs and perceptions of the healthcare system" - Please consider revising the word ordering of this text to improve readability.

*We thank the reviewer for this observation and have revised the manuscript accordingly. (Line 119)*

**3.56.**       Lines 104-105 - "In the wake of the Ebola epidemic, there has been significant investment and attention in health systems strengthening for the affected countries of West Africa" - It would be beneficial to the reader if some specific examples of how the health system has been strengthened were provided, particularly relating to Sierra Leone.

*We thank the reviewer for this observation and the following sentences have been added: “In the wake of the Ebola epidemic, there has been significant investment and attention in health systems strengthening for the affected countries of West Africa (23, 24). In April 2015, the Sierra Leone MoHS detailed a strategy to strengthen the health system which included, as its first key areas, establishing safe work settings and increasing the workforce (25). NGO’s in the area, such as Partners in Health, joined this objective working to improve the infrastructure of local health facilities (23). In Kenema specifically, a new 48 bed Lassa fever ward was built following the epidemic (26). While the desire to strengthen the health system is often the ‘dominant narrative’ after an epidemic, whether individuals are availing themselves of these services has not been adequately addressed in this setting (27).”(Lines 149-157)*

**3.57.**       Lines 107-108 - "HSB of patients with suspected Lassa fever" - The abstract did not specify that the recipients of the survey were patients with suspected Lassa fever; it only states that residents from 8 villages were surveyed. Please consider rewording to increase consistency throughout the manuscript.

*We thank the reviewer for this observation. The recipients of the survey were* ***not*** *patients with suspected Lassa fever, but rather asymptomatic individuals. We have revised the sentence as follows “This study sought to comprehend that knowledge gap by 1) assessing HSB of patients with suspected Lassa fever and 2) assessing general HSB of asymptomatic individuals through use of a questionnaire, in the Kenema district of Sierra Leone, a region that was severely impacted by Ebola.” (Lines 157-160)*

**3.58.**          Lines 113-114 - "While many individuals infected with LASV do not present to a health care provider or facility" - It is not clear if this is referring to the situation prior to or during/after the Ebola outbreak. The geographical scope of the statement is also unclear (i.e. Sierra Leone or Western Africa). Please reword to make this more explicit.

*We thank the reviewer for this observation and have revised the manuscript accordingly. (Lines 179-181)*

**3.59.**        Line 117 - "the Mastomys natalensis" - Please consider removing the "the" from this species reference.

*We agree with the reviewer and have made the suggested changes in the revised manuscript. (Line 184)*

**3.60.**          Line 121 - "hemorrhage" - I think this should be "hemorrhaging".

*We agree with the reviewer and have made the suggested changes in the revised manuscript. (Line 188)*

**3.61.**           Line 124 - Please confirm that reference #9 is the correct reference for this statement. Furthermore, the style of reference numbering has changed from parentheses to square brackets here and in the following line. Please revise for consistency.

*We thank the reviewer for this observation and have addressed these inconsistencies in the revised manuscript. (Line 191)*

**3.62.**          Line 124 - The abbreviation "LF" is used here for the first time. It should be first used immediately following the first use of the full expression "Lassa fever".

*We agree with the reviewer and have made the suggested changes in the revised manuscript.*

**3.63.**          Lines 127 - "quantify HSB for suspected Lassa fever" - Please revise this text to clarify the intended meaning. Furthermore, the abstract suggests that study's aim was to assess the impact of the Ebola outbreak on HSB, suggesting that the goal here should in fact be to quantify the change in HSB due to the Ebola outbreak. Please review and revise as necessary.

*We agree with the reviewer and have revised the sentence as follows: “The first goal of this study was to measure potential changes in HSB for suspected Lassa fever. Given the similarity in the diseases, HSB for Lassa fever may serve as a metric for the impact of the West African Ebola epidemic on HSB.”(lines 194-196)*

**3.64.**          Lines 128-129 - "an impact on HSB due to the Ebola epidemic" - This may be easier to read when worded as "the impact of the Ebola epidemic on HSB".

*We agree with the reviewer and have made the suggested changes in the revised manuscript. (Line 195-196)*

**3.65.**          Lines 129-130 - "Lassa endemic" - Please consider rewording to "Lassa fever endemic".

*We agree with the reviewer and have made the suggested changes in the revised manuscript. (Line 197)*

**3.66.**        Line 130 - "identify barriers" - Please consider rewording and/or providing examples to make meaning of "barriers" clearer.

*We agree with the reviewer and have made the suggested changes in the revised manuscript. (Lines 197-198)*

Summary Comments

**3.67.**       The paper makes it clear that presentations for Lassa Fever at KGH have declined post-Ebola outbreak compared to pre-Ebola outbreak, however, the connection between change in HSB and this decline in presentations is not made clear by the analysis presented, therefore the evidence and analysis provided do not sufficiently assess the impact of the Ebola outbreak on HSB with respect to presentations to KGH.

*We thank the reviewer for this observation and have added the following sentences to the revised manuscript: “A limitation of these results is that two data sets for different groups of subjects were used: one for asymptomatic individuals, and the other for symptomatic suspected Lassa fever cases. HSB cannot truly be measured until an individual experience’s symptoms. As such, we were unable to draw a direct linkage between the two datasets and the authors relied primarily on the surveillance data to draw conclusions.” (Lines 682-686)*

**3.68.**          The discussion does not fully evaluate how well the goals of the study have been achieved and provide detailed suggestions of how to better achieve the goals which were not fully addressed by the study.

*We thank the reviewer for this observation and have added into the discussion rhetoric around how well the goals of the study have been achieved, and suggestions as to how better to achieve the goals not fully address by the study. The following sentences have been added: “The goal of this study, to identify and evaluate the persistent impact of the Ebola epidemic on health seeking behavior for Lassa fever through hospital level data, and by survey questionnaire of general health seeking behavior, was met given the limitations of the methodology*

*The study identified a statistically significant decline in the number of individuals presenting to the hospital, or public health unit, with suspected Lassa fever. Although limited by sample size, the questionnaire of 194 individuals identified persistent reluctance to seek care for febrile illness since the Ebola epidemic among one fifth of respondents that could account for the decline noted at Kenema Government Hospital.*

*Identifying cause(s) beyond those self-reported for the decline in presentations at the hospital was beyond the scope of our study. What impact changes in community healthcare workers, transportation access, financial constraint or other potential drivers of HSB, had on this decline were not assessed in this study, but likely play an important role.*

*Further research into these, and potentially other, factors influencing underutilization of healthcare for Lassa fever is warranted.*

*To better achieve the goals of this study, a larger sample size, more villages, and more targeted questions regarding the interaction with public health units, and behavior more clearly linked to suspected Lassa fever could have strengthened the conclusions.*

*Additionally, targeting multiple aspects of the care pathway would have been valuable. For example, interviewing or analyzing community health workers, observing any changes in cost, distance, or time to care and other barriers identified by respondents in this paper.”(Lines 718-742)*

**3.69.**         The paper does not provide sufficient discussion of how this study's findings can be used to develop targeted public health strategies, particularly in Kenema.

*We thank the reviewer for this observation and have added into the discussion rhetoric around how the findings of the study can be used to develop public health strategies in Kenema. The following sentences have been added: “After an epidemic, or similar shock to the healthcare system and fabric of society, it is critical to be aware of the impact that has or does not have on morbidity, mortality, and healthcare access in communities. This research, and its findings are critical not only for management during the emergency, but in the years following.*

*This study confirms that health seeking behavior of individuals with suspected Lassa fever has not rebounded after the Ebola epidemic despite the maintenance of a state-of-the-art facility. These findings suggest that to optimize healthcare delivery post epidemic, investments into rebuilding the healthcare system and health of communities needs to be multifaceted. Beyond healthcare infrastructure, consideration of other important drivers of health seeking behavior should be considered.*

*Indirect effects on health and mortality during an epidemic are well known. However, factors that may persistently impact outcomes post pandemic may be more nuanced and less well studied or understood. Monitoring the impact on health seeking behavior (across health indicators) not only during an epidemic but in the years following is important.”(Lines 744-759)*
