# Supplementary material for: Health seeking behavior after the 2013–16 Ebola epidemic: Lassa fever as a metric of persistent changes in Kenema District, Sierra Leone
Source: PLoS Negl Trop Dis. 2021 Jul 14;15(7):e0009576. doi: 10.1371/journal.pntd.0009576 (PMC8312964; doi:10.1371/journal.pntd.0009576)
Supplement: Attachment — Submitted filename: PTND-D-20-01152_R3_ResponseToReviewers.docx [file pntd.0009576.s010.docx]

**POINT-BY-POINT REVIEWER RESPONSES**

Reviewer 3:

*Dear Reviewer 3,*

*We truly thank you for your careful review of our manuscript and constructive comments and have used this feedback to revise (and, we believe improve) the manuscript. Please find below our point-by-point italicized responses (including line numbers referencing the modification changes in the revised manuscript) to your critiques and comments. We do not believe that any of the changes in the revised manuscript altered the general conclusion or results presented in the original manuscript.*

Results

1.1. Lines 337-338 - "Moreover, individuals reported they were likely to seek care within 24 hours of symptoms arising (60%; Fig 4C)." - I recommend rewording such that the percentage is stated within the sentence to be consistent with the surrounding text. Furthermore, I recommend replacing "individuals" with "respondents", i.e 60% of respondents.

*We thank the reviewer for this comment and have made the necessary changes in the revised manuscript. (Line 345)*

1.2. Lines 339-341 - "72% of respondents..." and "78% of respondents..." - Please replace numerical figures with spelled out numbers when at the start of a sentence.

*We thank the reviewer for this observation and have made the necessary changes in the revised manuscript. (Lines 348-349)*

1.3. Lines 350-351 - "Self-reported satisfaction for health services was significantly higher among males than females ( [aOR] =2.1, [95% CI] = [0.87, 5.10]) (S7 Table, S8 Table)." - I recommend that the authors remove the expression "significantly" because the p-value for this result in S7 table is not statistically significant. Furthermore, this statement does not discuss perceived safety and therefore no reference to S8 Table is required.

*We thank the reviewer for this observation and have made the necessary changes in the revised manuscript. (Lines 358-359)*

1.4. Lines 351-352 - "Respondents were likely to say they received all the services they needed at their health facility (S8 Table)." - The results described by this sentence are not contained in S8 Table. Please revise the text and/or reference as necessary

*We thank the reviewer for this observation and have made the necessary changes in the revised manuscript. (Line 360)*

1.5. Lines 352-354 - "There were no other statistically significant differences amongst age groups; level of education and religious affiliation did not predict answers to either question." - S8 Table shows a p-value of less than 0.05 for the age group 37-45. Please revise the text accordingly to reflect the presence of this statistically significant variable.

*We thank the reviewer for this observation. While the p-value for the 37-45 year age group in Table S8 was significant (p = .042), its corresponding 95% confidence interval included one (0.92, 17.22). To address this discrepancy, we revised the statement as follows. There were no other statistically significant differences amongst age groups (with the exception of the 37-45 year age group, but its confidence interval included one); level of education and religious affiliation did not predict answers to either question.” (Lines 356-359).*

1.6. Lines 355-357 - "For this analysis, age categories, 18-27, 28-36, 37-45, and 45+, were selected to accommodate the age inclusion criteria for the HSB questionnaire." - This information would be more appropriately included in the Methods section.

*We agree with the reviewer and have moved the before-mentioned sentence into the Methods section. (Lines 241-242).*

Conclusions

1.7. Lines 409-410 - "Nigeria experienced their largest ever recorded LF outbreak" - Lassa fever is used in its abbreviated form here for the first time in the Discussion section with no prior definition. Furthermore, it is used in its full form several times prior to this sentence within the Discussion section. Please define after the first use and use the abbreviated version for subsequent uses.

*We thank the reviewer for this observation and have made the necessary changes in the revised manuscript.*

1.8. Line 426 - "one in five respondents" - I suggest adding "almost" to this statement because the actual figure is 22% according to S5 Table.

*We thank the reviewer for this comment and have made the necessary changes in the revised manuscript. (Line 453)*

1.9. Lines 449-450 - "Instead, HCW's empirically treated statistically significantly more individuals for malaria post- vs. pre- epidemic." - The meaning of this is not clear. If this is presenting a comparison to the previous sentence's statement then please more explicitly describe this connection. Furthermore, it would be more meaningful to provide the numeric value of this statistical significance.

*We thank the reviewer for this comment. The sentence in question has been reworded for increased clarity in the revised manuscript. Specifically, the sentence was reworded as: “However, a 2017 study assessing community HCW diagnosis in febrile patients in Kenema district found that rapid malaria testing (banned under no touch policies) had been replaced during the epidemic with empiric testing and had not yet returned to pre-epidemic levels (58). After the epidemic, HCW’s continued to presumptively treat significantly more individuals for malaria than pre-epidemic (p<.001). This change in HCW behavior for febrile illnesses may account for underreporting or decreased recognition of Lassa fever patients.” (Lines 470-476)*

1.10. Lines 468-469 - "...around 20% of respondents did not report increased trust in healthcare facilities. This group of 20%..." - I think it would be more accurate to directly refer to the survey answer of decreased attendance at Kenema hospital after the 2014 Ebola epidemic rather than the interpreted meaning of "increased trust in healthcare facilities". While this connection may be possible, the survey did not directly ask respondents about their trust in healthcare facilities. Furthermore, I would recommend replacing "This group of 20%" with "This group of respondents" because the group is not exactly 20%.

*We thank the reviewer for this comment and have made the necessary changes in the revised manuscript. (Lines 506-507)*

1.11. Lines 479-481 - "While others have reported differences in health seeking behavior among genders and age groups, our limited sample size was unable to identify an association." - Please be more explicit as to what is meant by "others". If this is referring to other studies then please provide supporting references

*We thank the reviewer for this comment. The sentence in question has been reworded for increased clarity in the revised manuscript. Specifically, the sentence was reworded as: “While gender and age have been reported to impact HSB in a variety of studies, we were unable to identify an association given the sample size (63-65).”* *(Lines 524-526)*

1.12. Line 481 - "Economic strength could be a factor, but was not assessed." - It is unclear what is meant by "economic strength". Please revise this sentence to better explain this factor

*We thank the reviewer for this comment. The sentence in question has been reworded for increased clarity in the revised manuscript. Specifically, the sentence was reworded as: “Financial resources of individuals or villages could be a factor, but was not assessed.” (Line 526)*

1.13. Lines 492-496 - "A limitation of these results is that two data sets for different groups of subjects were used: one for asymptomatic individuals, and the other for symptomatic suspected Lassa fever cases. HSB cannot truly be measured until an individual experience's symptoms. As such, we were unable to draw a direct linkage between the two datasets and the authors relied primarily on the surveillance data to draw conclusions." - The intended meaning of this paragraph is difficult to understand. I think that the fact that the study used data from two different sources is not a limitation in itself. While a direct linkage between the decline in LF presentations at KGH and changes in HSB could not be sufficiently assessed, this study was able to explore and identify some factors that led to initiation of HSB and factors that acted as barriers to HSB. Please revise this paragraph.

*We thank the reviewer for this comment. The sentence in question has been reworded for increased clarity in the revised manuscript. Specifically, the sentence was reworded as: “We present results from two data sets from two distinct groups of subjects in the Region: one of individuals in the general population and the other of symptomatic patients with suspected Lassa fever at KGH. While were unable to draw a direct linkage between the two data sets, we were able to identify some factors that may have facilitated and hindered HSB.”(Lines 537-540)*

1.14. Line 550 - "Indirect effects on health and mortality during an epidemic are well known." - Please add supporting references for these well-known effects.

*We thank the reviewer for this comment and have made the necessary additions in the revised manuscript. (Line 615)*

Introduction

1.15. Lines 112-114 - "Commonly used proxies for health seeking behavior have been healthcare consumption patterns, provider visits, and self-reported care seeking." - Please provide a reference to support this statement

*We thank the reviewer for this comment and have made the necessary additions in the revised manuscript. (Line 112-114)*

1.16. Lines 123-125 - "This study sought to comprehend that knowledge gap by 1) assessing HSB of patients with suspected Lassa fever and 2) assessing general HSB of asymptomatic individuals through use of a questionnaire, in the Kenema district of Sierra Leone, a region that was severely impacted by Ebola." - From my understanding after reading the entire manuscript, the HSB questionnaire was administered to 200 Sierra Leoneans across 8 villages in the Kenema district; however, the manuscript does not describe any specific selection of asymptomatic individuals for the survey. There may in fact have been asymptomatic individuals amongst those who participated the HSB survey; however, I would suggest removing the term "asymptomatic" from this sentence to avoid confusion.

*We thank the reviewer for this comment and have made the necessary changes in the revised manuscript. (Line 124)*
